# Dissecting the Molecular Mechanism of Wang-Bi Capsule in the Treatment of Experimental Rheumatoid Arthritis Based on Synovial Tissue Proteomic Analysis

**DOI:** 10.1155/2021/5539008

**Published:** 2021-10-18

**Authors:** Haiyang Shu, Yingjie Shi, Li Li, Ning Zhao, Cheng Lu, Aiping Lu, Xiaojuan He

**Affiliations:** ^1^Institute of Basic Research in Clinical Medicine, China Academy of Chinese Medical Sciences, Beijing 100700, China; ^2^The Second Clinical College of Guangzhou University of Chinese Medicine, Guangzhou 510006, China; ^3^Shanghai Innovation Center of TCM Health Service, Shanghai University of Traditional Chinese Medicine, Shanghai, China; ^4^Law Sau Fai Institute for Advancing Translational Medicine in Bone & Joint Diseases, School of Chinese Medicine, Hong Kong Baptist University, Kowloon Tong, Hong Kong; ^5^Shanghai Guanghua Hospital of Integrated Traditional Chinese and Western Medicine, Institute of Arthritis Research, Shanghai Academy of Chinese Medical Sciences, Shanghai, China; ^6^Academy of Integrative Medicine, Shanghai University of Traditional Chinese Medicine, Shanghai 201203, China

## Abstract

Wang-Bi capsule (WB) is a traditional Chinese medicine formula and has been applied for rheumatoid arthritis (RA) treatment for many years. However, its underlying molecular mechanisms still remain unclear. In this study, collagen-induced arthritis (CIA) rats were used to observe the therapeutic effect of WB used at different time points, and the proteomic analysis of synovial tissue was applied to reveal its basic molecular mechanisms. The results demonstrated that WB not only effectively ameliorated the symptoms and synovitis, but also downregulated the serum levels of inflammatory cytokines/chemokines in CIA rats. Furthermore, the proteomic analysis of synovial tissue showed that WB could regulate several signaling pathways associated with inflammation or cell migration, such as “IL-1 signaling,” “IL-8 signaling,” and “CXCR4 signaling.” The expression levels of proteins including matrix metalloproteinase 3 (MMP3), MMP19, lipopolysaccharide-binding protein (LBP), serine/threonine kinase interleukin-1 receptor-associated kinase 4 (IRAK4), and actin-related protein 2/3 complex subunit 5 (ARPC5) in these pathways were downregulated significantly by WB when compared with the model group. In sum, this study indicated that WB had obvious inhibitory effects on synovitis of CIA rats, and the mechanisms of which may be involved in downregulating the expression levels of several key proteins including MMP3, MMP19, LBP, IRAK4, and ARPC5.

## 1. Introduction

Rheumatoid arthritis (RA) is a common chronic inflammatory disease with high incidence and disability rate, and one of the main pathological characteristics of it is the inflammation of the synovial membrane [1, 2]. Synovial cells release various inflammatory mediators, such as interleukin-1*β* (IL-1*β*), tumor necrosis factor *α* (TNF-*α*), and reactive oxygen species (ROS), which are both initiators of inflammatory response and important regulatory mediators in the development of disease [3]. Inflammation also leads to the excessive hyperplasia of synovial cells [4]. Moreover, synovial fibroblast-like cells (FLS) in synovial tissues can promote the production of receptor activator of nuclear factor *κ*B ligand (RANKL) and matrix metalloproteinases (MMPs), which aggravate joint damage. RANKL can activate macrophages to differentiate into osteoclasts, which mediates bone damage via promoting bone resorption. MMPs are important enzymes for cartilage degradation. Therefore, it is an important therapeutic strategy to inhibit synovitis in RA. It is known to all that synovitis mainly results from leukocyte migration and infiltration of the synovial compartment, which is mediated by abundant of adhesion molecules and chemokines [5, 6]. Amounts of infiltrated cells including granulocytes, monocytes/macrophages, B cells, and T cells have been found in the synovial tissue of patients with RA [7–10]. These infiltrated cells secrete plentiful proinflammatory cytokines which can further aggravate inflammation response of synovium. Therefore, it is important to inhibit the migration of immune cells into synovial membrane.

Although RA is an uncurable disease currently, many effective therapies have been made during the past two decades [11]. Patients have benefited from some nonsteroidal anti-inflammatory drugs, disease-modifying antirheumatic drugs, and several biological agents [12, 13]. However, these therapy options still cannot meet all patients, because of insufficient response or adverse events [14, 15].

Wang-Bi capsule (WB), approved by China Food and Drug Administration (approval ID: Z20080096), is a traditional Chinese medicine formula consisting of seventeen herbs. As a complementary and alternative drug, WB has been used in RA treatment for many years and achieved a good note in efficacy and safety. WB could downregulate the production of proinflammatory cytokines including TNF-*α*, IL-6, and IL-17*α* and upregulate the anti-inflammation cytokines like IL-10 in the serum of collagen-induced arthritis (CIA) rats [16, 17]. In our previous study, we found that WB could ameliorate arthritis symptoms in CIA mice. Moreover, it could alleviate the synovial inflammation and bone destruction via modulating the OPG/RANKL system and inhibiting the activation of NF-*κ*B [18]. Simultaneously, Icariin, one of the active ingredients of WB, was proved to inhibit cell migration or invasion via regulating MMP1/3 expression [19]. Paeonol is also an effective ingredient of WB, which could decrease the expression levels of MMP1/3 in rats with CIA [20]. Paeoniflorin is one ingredient of WB, which could ameliorate inflammatory cell infiltration including macrophages and neutrophils via suppressing RhoA/ROCK signaling [21, 22]. However, the precise therapeutic effect and pharmacological mechanisms of WB on RA, especially about inflammation and cell migration, still need further investigation. Recently, isobaric tag for relative and absolute quantification (iTRAQ) has become an important method to study the mechanism of action of complicated herb formula. At the same time, bioinformatics analysis has also become a good tool to explore the potential mechanism of the drugs and the signaling pathways/networks from the large number of data generated by omics [23, 24]. Therefore, in this study, we not only observed the therapeutic effect of WB administrated at different time points in CIA rats, but also explored its molecular mechanisms by using proteomic analysis of synovium tissue, which hope to present fundamental research evidence for rational use of WB.

## 2. Materials and Methods

### 2.1. Animals

Male Wistar rats (6-8 weeks) were supplied by the Vital River Laboratory Animal Technology Co., Ltd. (Beijing, China) and housed in specific pathogen free conditions (25 ± 2°C, humidity: 60 ± 10%, 12 h light : 12 h dark) with free access to sterilized water and chow. The study was conducted in line with the Guide for Care and Use of Laboratory Animals and approved by the Research Ethics Committee of Institute of Basic Theory of Chinese Medicine, China Academy of Chinese Medical Sciences.

### 2.2. Establishment of CIA Model

The CIA model was established according to the previous reports [22]. Bovine type II collagen (Chondrex, Redmond, WA, USA) was emulsified with equal volume incomplete Freund's adjuvant (Chondrex, Redmond, WA, USA) at 4°C; then, the rats received a subcutaneous injection of 200 *μ*l emulsion prior prepared at the base of the tail, and another 100 *μ*l emulsion was injected on day 7 in the same manner for the boost immunization.

### 2.3. Grouping and Treatment

**Figure 1:**
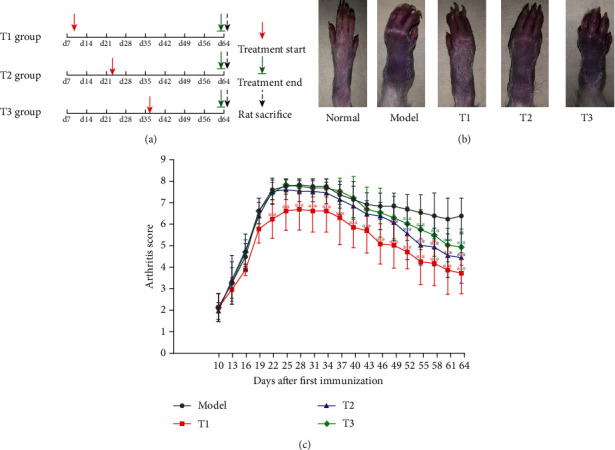
WB ameliorated the symptoms of CIA rats. (a) The regimen of treatment. (b) The photographs of hind paw from different groups at day 64. (c) Arthritis score. Data were mean ± SD. ^∗∗^*P* < 0.01 and ^∗^*P* < 0.05 compared with the model group.

WB was purchased from Liaoning China Resources Benxi Sanyao Co., Ltd. (Liaoning, China, No. 20180205). The drug samples were characterized by high-performance liquid chromatography (HPLC) fingerprinting analysis (Supplementary Fig. 1). The drug was dissolved in double-distilled water and was totally blended again prior to use. Rats were randomly divided into five groups after the successful induction of the CIA model: normal group, model group, the first treatment group (T1 group), the second treatment group (T2 group), and the third treatment group (T3 group). The rats in each treatment group were administrated by gavage with WB (0.74 g/kg/d, the dose corresponding for RA patients) daily starting on day 10 or day 23 or day 36 after the first immunization, respectively. The rats in the normal group and model group were administered the corresponding volume of double-distilled water. The regimen of treatment is described in Figure 1(a). All the animals were sacrificed on day 65 after the first immunization. Serums were harvested for multifactor detection assay. The knee joints were removed for histological analysis. The synovial tissue separated from the knee joints was used for proteomic analysis and western blot.

### 2.4. Assessment of Arthritis Score

The severity of arthritis in CIA rats was scored every three days, and the arthritis score of hind limbs was graded for severity of swelling and redness using a macroscopic scoring system: 0 point for no redness and swelling, 1 point for slight swelling and/or erythema, 2 points for low-to-moderate edema, 3 points for pronounced edema with limited joint usage, and 4 points for excess edema with joint rigidity. The maximum arthritis score of each rat was eight [22].

### 2.5. Multifactor Assay

Cytokines and chemokines in rat's serum samples were measured using a 22-plex kit (EBIO Procarta Plex Panel, USA), and the assay was performed according to the protocol. All samples were assayed in duplicate and analyzed with a Luminex 200 Labmap system (Luminex, USA).

### 2.6. Histopathological Analysis

The knee joints of rats were fixed in 10% phosphate buffered formalin for 3 days and then were decalcified in 10% EDTA. Tissue sections were stained with hematoxylin and eosin (H&E). The feature score of synovium was scored on a scale of 0~9 (including hyperplasia of synovial lining cell layer, infiltration of inflammatory cells, and pannus formation) [25].

### 2.7. Proteomic Analysis

iTRAQ proteomics was applied to analyze synovial tissue of the knee joint from the normal group, model group, and T1 group. The protein extraction, quality control, and proteolysis were performed according to the protocol. The iTRAQ® reagent 8 plex (AB Sciex Pte. Ltd., USA) was used to label peptides. The Shimadzu LC-20AB liquid phase system (Shimadzu, Japan) was used for peptide fractionation, and peptide samples were separated by Thermo UltiMate 3000 UHPLC (Thermo Fisher Scientific, San Jose, USA). The peptides separated by liquid phase chromatography were ionized and then passed to a tandem mass spectrometer Q-Exactive HF X (Thermo Fisher Scientific, San Jose, CA, USA) for data-dependent acquisition (DDA) mode detection.

The raw mass spectrometry (MS) data was converted into Mascot Generic Format (MGF) format, which was then searched by the local Mascot server. In addition, quality control was performed, and IQuant soft was applied to the quantification of proteins [26]. To assess the confidence of peptides, the Professional Scrum Master™ levels (PSMs) were prefiltered at a PSM-level false discovery rate (FDR) of 1%. The protein FDR was also set at 1% (protein − level FDR ≤ 0.01).

### 2.8. Ingenuity Pathway Analysis (IPA)

Proteins with 1.2 fold change (mean value of all comparison groups) and *P* value (*t*-test of all comparison groups) less than 0.05 were defined as differentially expressed proteins (DEPs). The ingenuity pathway analysis (IPA) software was used to evaluate the trend of protein enrichment changes. The DEPs with fold changes were inputted into the IPA software, and then, the function annotations, ingenuity canonical pathways, and pathway networks were algorithmically generated according to protein-protein interactions.

### 2.9. Western Blot

Synovial tissue was lysed for extracting protein according to standard method, and 15 *μ*g of total protein was separated on denaturing 12% polyacrylamide gels and transferred to immunoblot polyvinylidene fluoride (PVDF) membrane (Millipore, Germany). Membranes were incubated in Tris-buffered saline with 0.1% Tween-20 containing 5% skim milk for 0.5 h at room temperature. Blots were probed with primary antibodies to MMP3 (1 : 1000, CST, USA), MMP19 (1 : 1000, Proteintech Group, Inc., USA), lipopolysaccharide-binding protein (LBP) (1 : 1000, Abcam, USA), Arp2/3 complex 5 (ARPC5) (1 : 1000, Invitrogen, USA), interleukin-1 receptor-associated kinase 4 (IRAK4) (1 : 1000, CST, USA), and *β*-actin (1 : 1000, Beyotime Biotechnology, China) overnight at 4°C. After extensive washes, membranes were probed with the appropriate HRP-conjugated secondary antibodies (1 : 1000, Beyotime Biotechnology, China) and washed extensively with Tris-buffered saline. Signals were detected using the Luminata Classico Western HRP Substrate (Millipore, Germany) and imaged using a Chemi Doc Touch Imaging System (Bio-Rad, USA).

### 2.10. Statistical Analysis

The GraphPad prism v.6 (GraphPad Software, San Diego, CA, USA) was used for the statistical analyses. The Student *t*-test was used for the analysis of differences between two groups, and the ANOVA was performed for comparisons among the groups. The data in this research was presented as mean ± SD, and statistical significance was accepted at *P* value <0.05.

## 3. Results

### 3.1. WB Effectively Ameliorated the Symptoms of CIA Rats

To determine the effect of WB on CIA rats, administration of WB started at day 10 or day 23 or day 36 after first immunization, respectively (Figure 1(a)). The hind paw features including swelling and malformation were markedly observed in rats of the model group, but the severity of which was relieved by some degrees in WB treatment groups (T1, T2, and T3 groups) at day 64 compared with the model group (Figure 1(b)). Consistently, the arthritis score was also lowered significantly after WB treatment compared with the model group (Figure 1(c)). In addition, there was no significantly hepatotoxicity and nephrotoxicity after the administration of WB (Supplementary Fig. 2).

### 3.2. WB Inhibited the Joint Inflammation of CIA Rats

**Figure 2:**
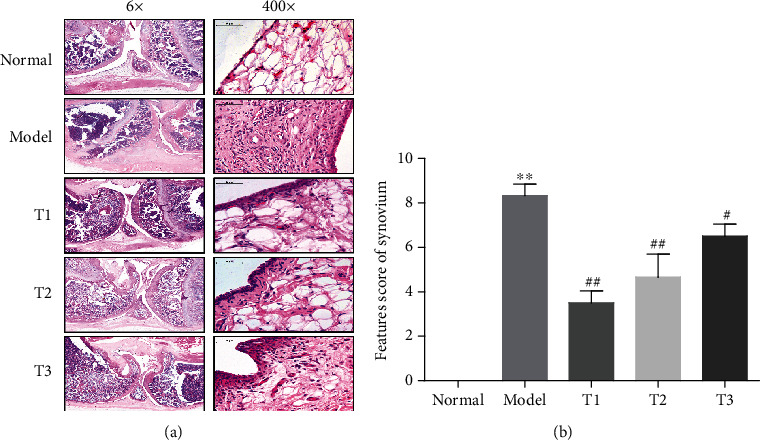
WB alleviated the joint inflammation of CIA rats. (a) Representative histopathological finding of knee joint from each group. The sections of knee joint were stained with H&E (magnification 6x and 400x). (b) Features score of synovium from each group. Data were mean ± SD, *n* = 6. ^∗∗^*P* < 0.01 compared with the normal group; ^##^*P* < 0.01 and ^#^*P* < 0.05 compared with the model group.

To further assess the effect of WB on CIA rats, histopathological features of the knee joints were observed. Compared with the normal group, the model group showed aggressive expansion of synovial membrane and more infiltration of inflammatory cells. WB treatment (T1, T2, and T3 groups) could inhibit the expansion of synovial membrane and relieve the infiltration of inflammatory cells in varying degrees compared with the model group (Figure 2).

### 3.3. WB Decreased the Levels of Serum Inflammatory Cytokines/Chemokines in CIA Rats

**Figure 3:**
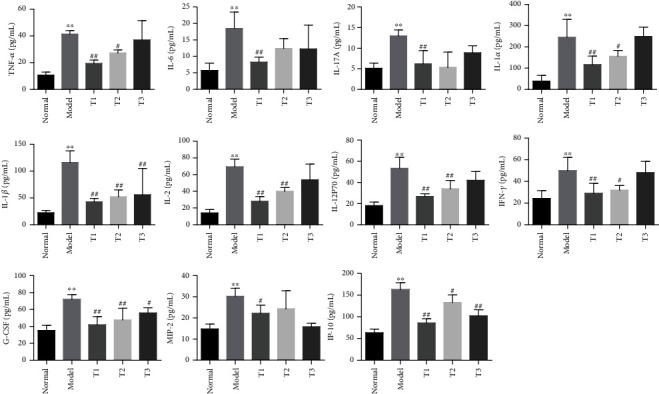
WB downregulated the serum levels of cytokines/chemokines in CIA rats. Multifactor detection of TNF-*α*, IL-6, IL-17A, IL-1*α*, IL-1*β*, IL-2, IL-12P70, IFN-*γ*, G-CSF, MIP-2, and IP-10 in serum of rats from different groups. Data are mean ± SD, *n* = 6. ^∗∗^*P* < 0.01 compared with the normal group; ^##^*P* < 0.01 and ^#^*P* < 0.05 compared with the model group.

To further investigate the therapeutic effects of WB on CIA rats, the concentration of inflammatory cytokines and chemokines in serum was measured by multifactor detection kit. The inflammatory cytokines such as TNF-*α*, IL-6, IL-17A, IL-1*α*, IL-1*β*, IL-2, IL-12P70, IFN-*γ*, and G-CSF as well as some chemokines like MIP-2 and IP-10 were significantly upregulated in the model group compared with the normal group. However, the levels of these mediators were shown to have a remarkable downregulation in the T1 group (in the T2 group or the T3 group, some but not all mediators were significantly downregulated) when compared with the model group (Figure 3).

### 3.4. Function Enrichment of DEP Analysis

**Figure 4:**
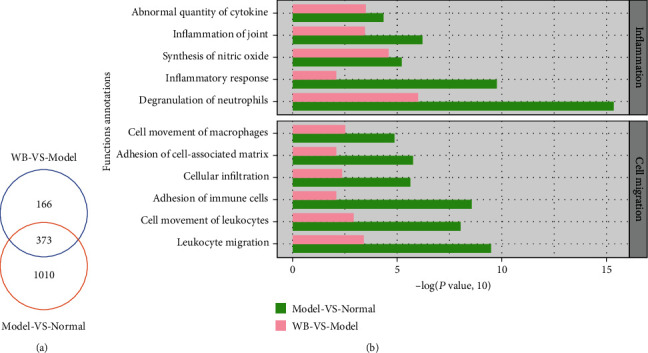
The biofunction analysis of DEPs in WB vs. model and model vs. normal. (a) The Venn diagram of DEPs of WB vs. model and model vs. normal. (b) The top function enrichment annotations in inflammation and cell migration were screened with −log(*P* value, 10) ≥ 1.3.

The synovial tissue of knee joint in the normal group, model group, and T1 group was processed for proteomic analysis. 1383 DEPs were detected in model vs. normal, and 539 DEPs were detected in WB vs. model, and 373 shared DEPs were detected in two paired groups (protein fold change ≥ 1.2, *P* value ≤0.05) (Figure 4(a)). To assess the general features of DEPs altered by WB in synovial tissue, 1383 DEPs in model vs. normal and 539 DEPs in WB vs. model were, respectively, inputted into the IPA software, and the function enrichment (−log(*P* value, 10) ≥ 1.3) lists were output. As inflammation and leukocyte infiltration played the central roles in RA synovitis progression, the top function enrichment annotations associated with inflammation and cell migration were further concerned in this study. Top function enrichment annotations of inflammation were “abnormal quantity of cytokine,” “inflammation of joint,” “synthesis of nitric oxide,” “inflammation response,” and “degranulation of neutrophils.” And top function enrichment annotations of cell migration were “cell movement of macrophages,” “adhesion of cell-associated matrix,” “cellular infiltration,” “adhesion of immune cells,” “cell movement of leukocytes,” and “leukocyte migration” (Figure 4(b)).

### 3.5. Pathway/Network Analysis

**Figure 5:**
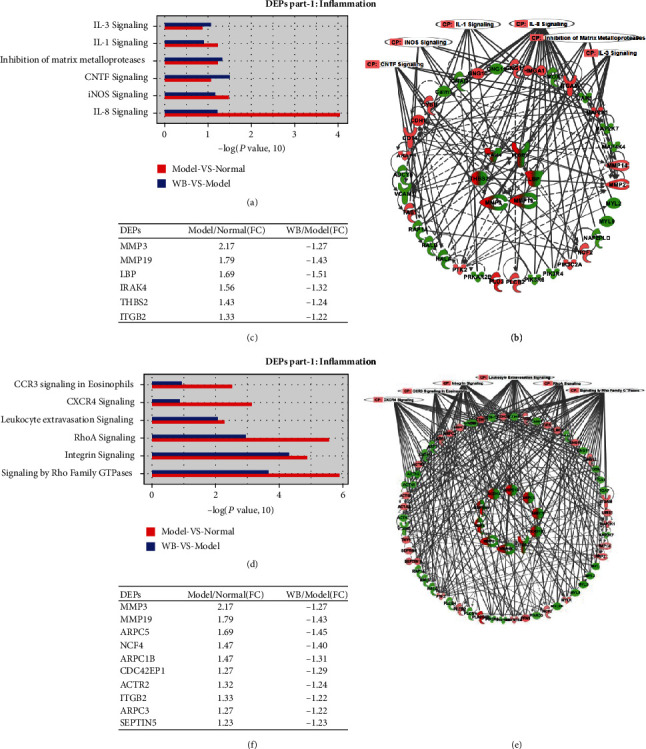
The pathway and network analysis of DEPs in WB vs. model and model vs. normal. (a, d) The ingenuity canonical pathway enrichment analysis of DEPs part 1 and DEPs part 2 was analyzed, and the threshold was set as −log(*P* value, 10) > 0.85. (b, e) The protein-protein interaction network of DEPs part 1 and part 2 were generated, respectively. (c, f) The fold changes of shared DEPs in inflammation network and cell migration network were listed, respectively. Note: in (b) and (e), the outer rings were unshared DEPs in model vs. normal or WB vs. model, pink color represented upregulation of protein expression, and green color represented downregulation of protein expression. The inner rings were shared DEPs, red color represented upregulation of protein expression in model vs. normal, and green color represented downregulation of protein expression in WB vs. model.

To further explore how WB regulated the inflammation and cell migration, the ingenuity canonical pathways were analyzed and the threshold was set as −log(*P* value, 10) > 0.85. Top inflammation pathways were “IL-3 signaling,” “IL-1 signaling,” “inhibition of matrix metalloproteases,” “CNTF signaling,” “iNOS signaling,” and “IL-8 signaling” (Figure 5(a)). Top cell migration pathways were “CCR3 signaling in eosinophil,” “CXCR4 signaling,” “leukocyte extravasation signaling,” “RhoA signaling,” “integrin signaling,” and “signaling by Rho family GTPases” (Figure 5(d)). Two networks were generated, respectively, with the two parts of DEPs: inflammation network (DEP part 1) and cell migration network (DEP part 2). In the inflammation network (Figure 5(b)), DEPs including MMP3, MMP19, LBP, and IRAK4 were upregulated in model vs. normal but downregulated in WB vs. model. The fold changes of these DEPs were listed (Figure 5(c)). In the cell migration network (Figure 5(e)), DEPs including MMP3, MMP19, and ARPC5 were upregulated in model vs. normal but downregulated in WB vs model. The fold changes of these DEPs were listed (Figure 5(f)).

### 3.6. Validation of DEPs

**Figure 6:**
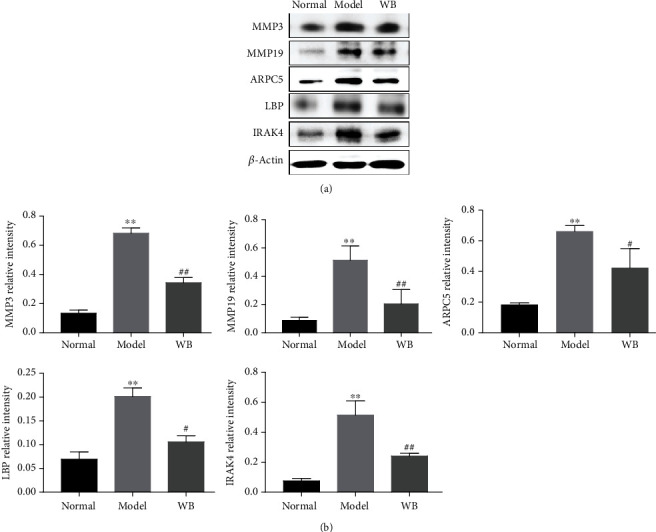
The validation of DEPs. (a) Representative bands of western blot in different groups. (b) Semiquantitative analysis of western blot in different groups. Data were mean ± SD, *n* = 3. ^∗∗^*P* < 0.01 compared with the normal group; ^##^*P* < 0.01 and ^#^*P* < 0.05 compared with the model group.

The shared DEPs (model vs. normal and WB vs. model) with a fold change > 1.5 in inflammation network and cell migration network were chosen to validate by western blot assay. The expression levels of proteins (MMP3, MMP19, LBP, IRAK4, and ARPC5) in the model group were significantly upregulated when compared with the normal group, whereas the levels of these proteins were remarkably downregulated in WB group when compared with the model group (Figure 6 and Supplementary Fig. 3).

## 4. Discussion

Although early diagnosis and treatment could effectively slow progression of disability, many patients with RA fail to deserve suitable drugs in time, because of nonresponse or adverse reactions [1]. In this study, we found that administration of WB at different time points could improve the arthritic symptoms of CIA rats. The histopathological assay and multifactor detection showed that WB administrated at different time points could remarkably alleviate the synovitis and decrease the levels of inflammatory factors in peripheral blood of CIA rats. Additionally, the sooner WB was administrated, the more benefit the CIA rats would get.

As well known, the hallmark of RA is perpetuation of synovial inflammation. The synovial membrane plays a central role in RA pathologic process. In our previous study, we found WB could effectively relieve the synovitis in CIA mice [18], but the exact mechanism has not been investigated thoroughly. Therefore, the synovial membranes of knee joints in the normal group, the model group, and the first treatment group (T1 group) were collected for further proteomic analysis in this study. And DEPs that were significantly enriched in two categories of functions (“inflammation” and “cell migration”) in terms of synovial inflammation were paid attention.

DEPs contributing to “inflammation” or “cell migration” category functions were complex, so pathway analysis was performed. We found that DEPs contribute to “inflammation” category signaling pathways including “iNOS signaling,” “IL-1 signaling,” “IL-8 signaling,” “inhibition of matrix metalloproteases,” “IL-3 signaling,” and “CNTF signaling.” The iNOS signaling mediates many inflammation or immune response. Overproduction of NO due to iNOS signaling activation leads to T cell or vascular endothelial cell dysfunction, which plays a key role in pathogenesis of RA [2, 27, 28]. IL-1 plays a crucial role in both inflammation response and bone destruction in RA [29]. Blockers that inhibit IL-1 signaling can significantly improve clinical and histological disease parameters of patients with RA [30]. IL-8 signaling contributes to anticitrullinated protein antibody- (ACPA-) mediated bone loss [31]. In addition, IL-8 signaling is also supposed to facilitate ACPA-induced joint inflammation via a chemokine-dependent manner [32]. Furthermore, IL-8 can promote synovial tissue-derived fibroblast migration and invasion into bone [33]. MMPs have also been well characterized in RA. Inhibition of MMPs can contribute to RA treatment [34]. IL-3 signaling plays important role in pathology of chronic inflammation [35]. And ciliary neurotrophic factor (CNTF) is one of IL-6 family cytokines, which contributes to synovitis [36]. Therefore, directly inhibition of inflammation response may be one of mechanisms that WB treats RA. In addition, WB controlling cell migration (such as macrophages and leukocytes) may boost its anti-inflammation effect. The DEPs contribute to “cell migration” category pathways including “CCR3 signaling in eosinophil,” “CXCR4 signaling,” “leukocyte extravasation signaling,” “RhoA signaling,” “integrin signaling,” and “signaling by Rho family GTPases.” These pathways consist an intricate network involved in inflammatory cell migration. CCR3 signaling is important for CXCL10 regulation inflammation responses via the activation and recruitment of leukocytes, like T cells and monocytes [37, 38]. CXCR4 signaling, as a member of CXCL12-CXCR4 axis, could promote cell migration and invasion [39]. Suppression of CXCR4 signaling significantly inhibited the invasion of FLS [40]. Activation of leukocyte extravasation or integrin signaling can directly contribute to infiltration of the synovial membrane with inflammatory cells. Both RhoA signaling and signaling by Rho family GTPases participate cell migration or invasion [41–43].

To further investigate the protein targets of that WB regulated inflammation and cell migration, the protein-protein interaction networks were generated. The results showed that WB could inhibit the expression of proteins including MMP3, MMP19, LBP, IRAK4, and ARPC5. MMP3, known as a reliable marker for RA activity and joint destruction [44], was produced by synovial fibroblasts and stimulated vascular permeability to enable leukocyte or monocyte extravasation from vessels at inflamed site [45, 46]. The areas of neutrophil accumulation were decreased in MMP-3^−/−^ mice compared with wild-type mice, and it was supposed that MMP3 could directly damage the connective tissue vessel wall [47]. MMP19 promoting neutrophil migration was also detailed in many researches to date [48]. LBP, a biomarker to evaluate RA disease activity [49], could induce macrophage activation [50, 51]. Both systematic and local LBP burden were associated with the abundance of active macrophages in joint capsule and synovium [52]. IRAK4 is an important node for mediating the IL-1/8 signaling, aberrant activation of which could trigger the inflammatory cascade and further lead to increased autoimmune response in synovium tissue [53–56]. Arp2/3 is required for the leukocytes rapidly and effectively moving [57, 58]. Arp2/3-deficient cells exhibit significantly slower migration compared with that of the control [59]. ARPC5, as one part of Arp2/3 complex, was identified to contribute to cell migration and invasion in many carcinomas [60]. Although the function of ARPC5 in RA has not been described in detail to date, our study suggested that ARPC5 might be one of the potential targets for WB treatment. These results also provided new targets for the development of novel RA drugs.

In conclusion, this study demonstrated that WB could effectively inhibit the synovial inflammation of CIA rats; the molecular mechanism was partly via downregulating the expression levels of several key proteins (MMP3, MMP19, LBP, IRAK4, and ARPC5). This study may provide a basic research evidence for rational use of WB in clinic.

## Data Availability

The data used to support the findings of this study are available from the corresponding authors upon request.

## Supplementary Materials

Supplementary Materials Supplementary Figure 1: high-performance liquid chromatography profile of WB at 235 nm. Supplementary Figure 2: the serum concentrations of AST, ALT, CREA, and UREA in CIA rats. Data were mean ± SD, *n* = 6. Note: NS: no significant difference. Supplementary Figure 3: original western blot images.
