# Supplementary material for: Dissecting the Molecular Mechanism of Wang-Bi Capsule in the Treatment of Experimental Rheumatoid Arthritis Based on Synovial Tissue Proteomic Analysis
Source: J Immunol Res. 2021 Oct 18;2021:5539008. doi: 10.1155/2021/5539008 (PMC8545597; doi:10.1155/2021/5539008)
Supplement: Supplementary Materials — Supplementary Figure 1: high-performance liquid chromatography profile of WB at 235 nm. Supplementary Figure 2: the serum concentrations of AST, ALT, CREA, and UREA in CIA rats. Data were mean ± SD, n = 6. Note: NS: no significant difference. Supplementary Figure 3: original western blot images. [file 5539008.f1.docx]

Supplementary Material


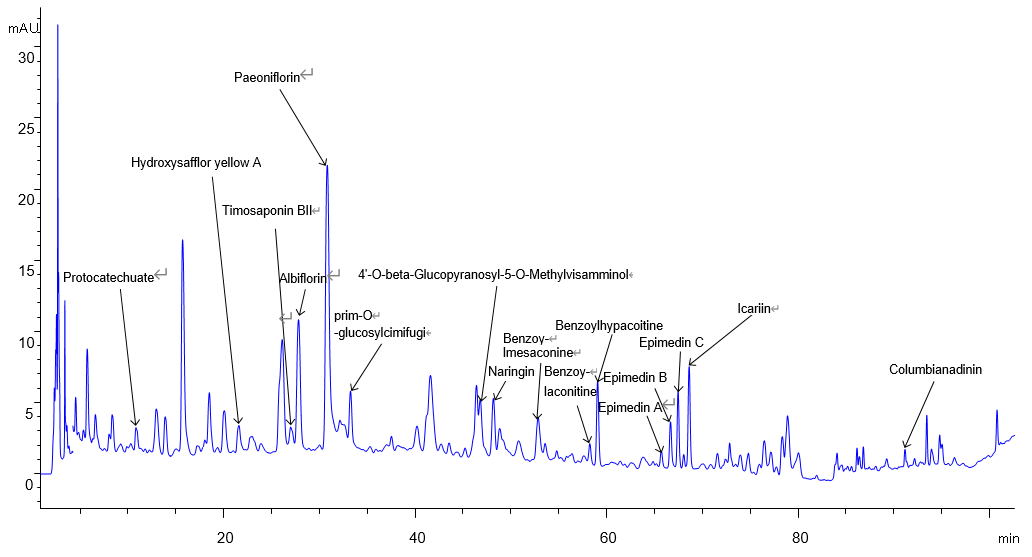


**Supplementary Figure 1: High‑performance liquid chromatography profile of WB at 235 nm.**

**
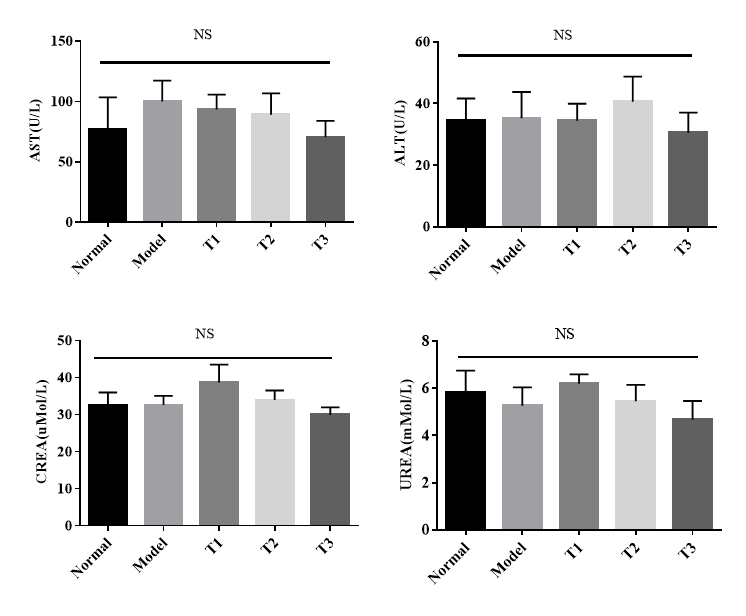
 Supplementary Figure 2: The serum concentrations of AST, ALT, CREA and UREA in CIA rats.** Data were mean ± SD, n=6. Note: NS: no significant difference.

1. **MMP3**

**Nor1# Mod1# WB1# | Nor2# Mod2# WB2# | Nor3# Mod3# WB3#**


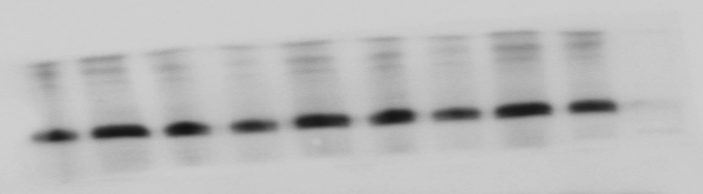


60kDa

1. **MMP19**

**Nor1# Mod1# WB1# |Nor2# Mod2# WB2# |Nor3# Mod3# WB3#**


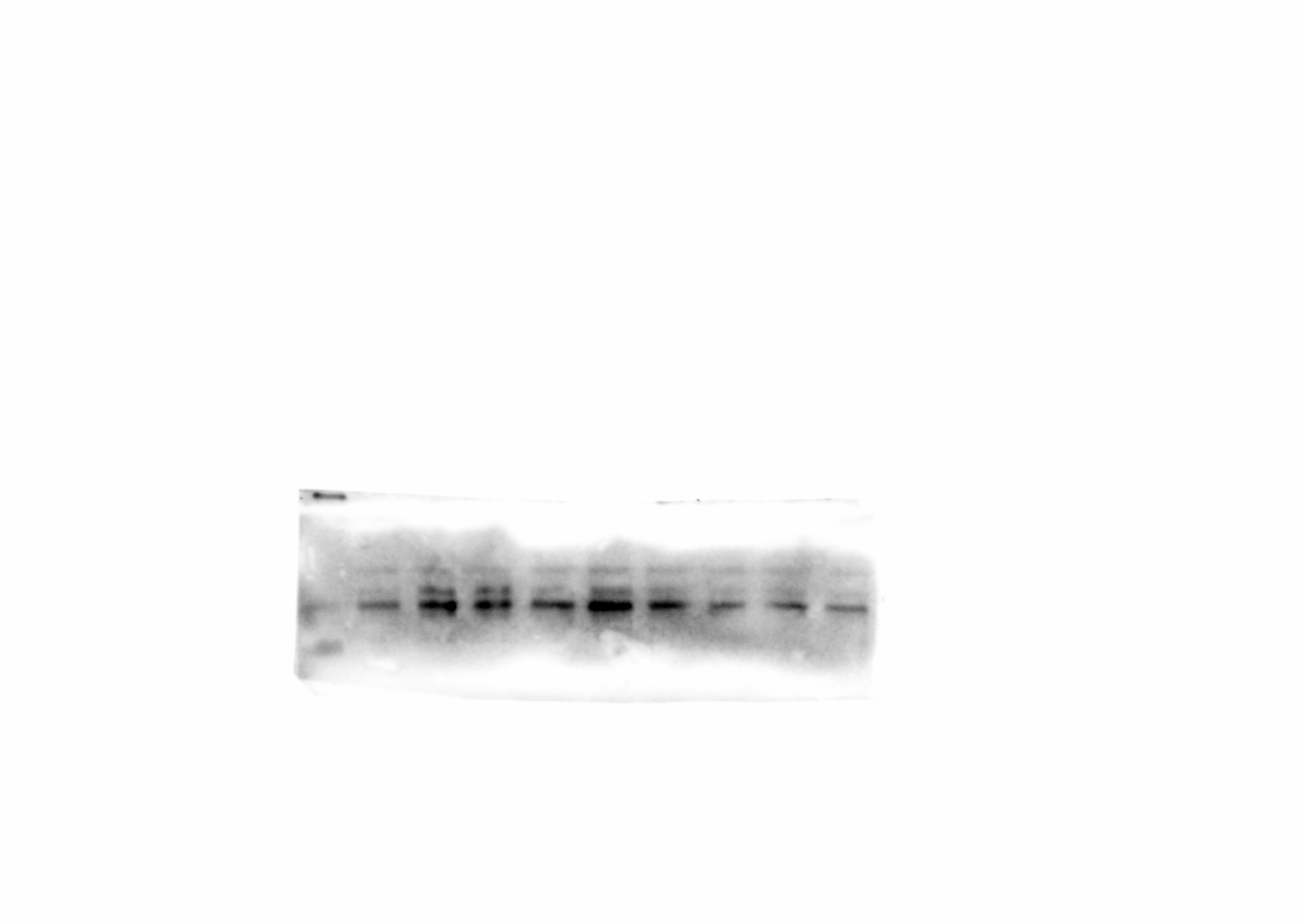


60kDa

1. **ARPC5**

**Nor1# Mod1# WB1# | Nor2# Mod2# WB2# | Nor3# Mod3# WB3#**


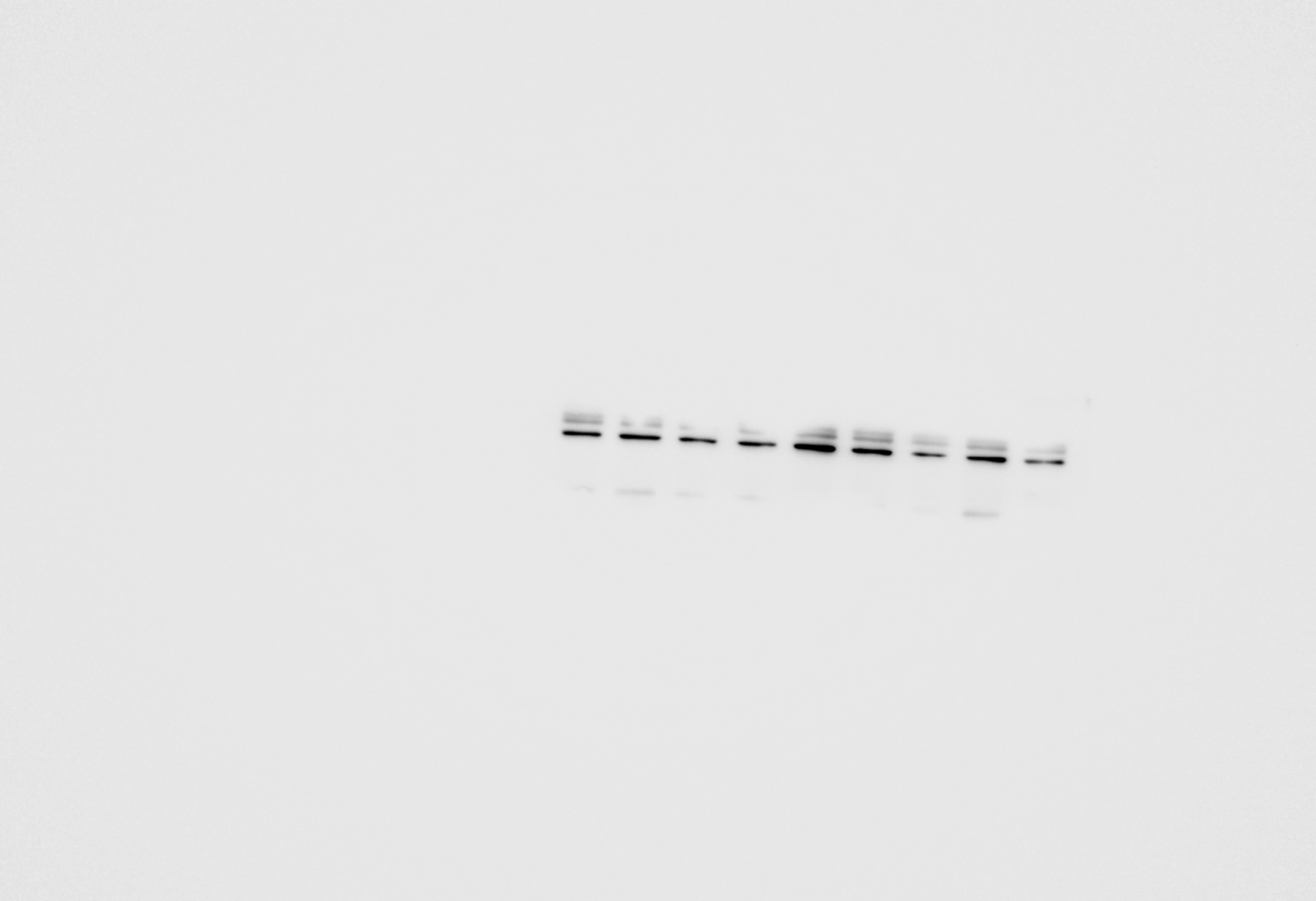


15kDa

1. **LBP**

**Nor1# Mod1# WB1# |Nor2# Mod2# WB2# |Nor3# Mod3# WB3#**


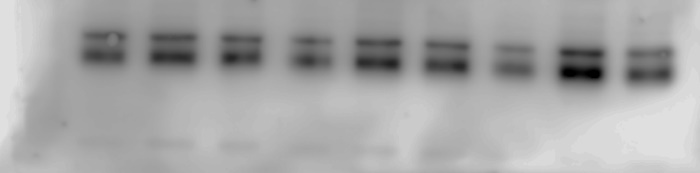


65kDa

1. **IRAK4**

**Nor1# Mod1# WB1# | Nor2# Mod2# WB2# |Nor3# Mod3# WB3#**


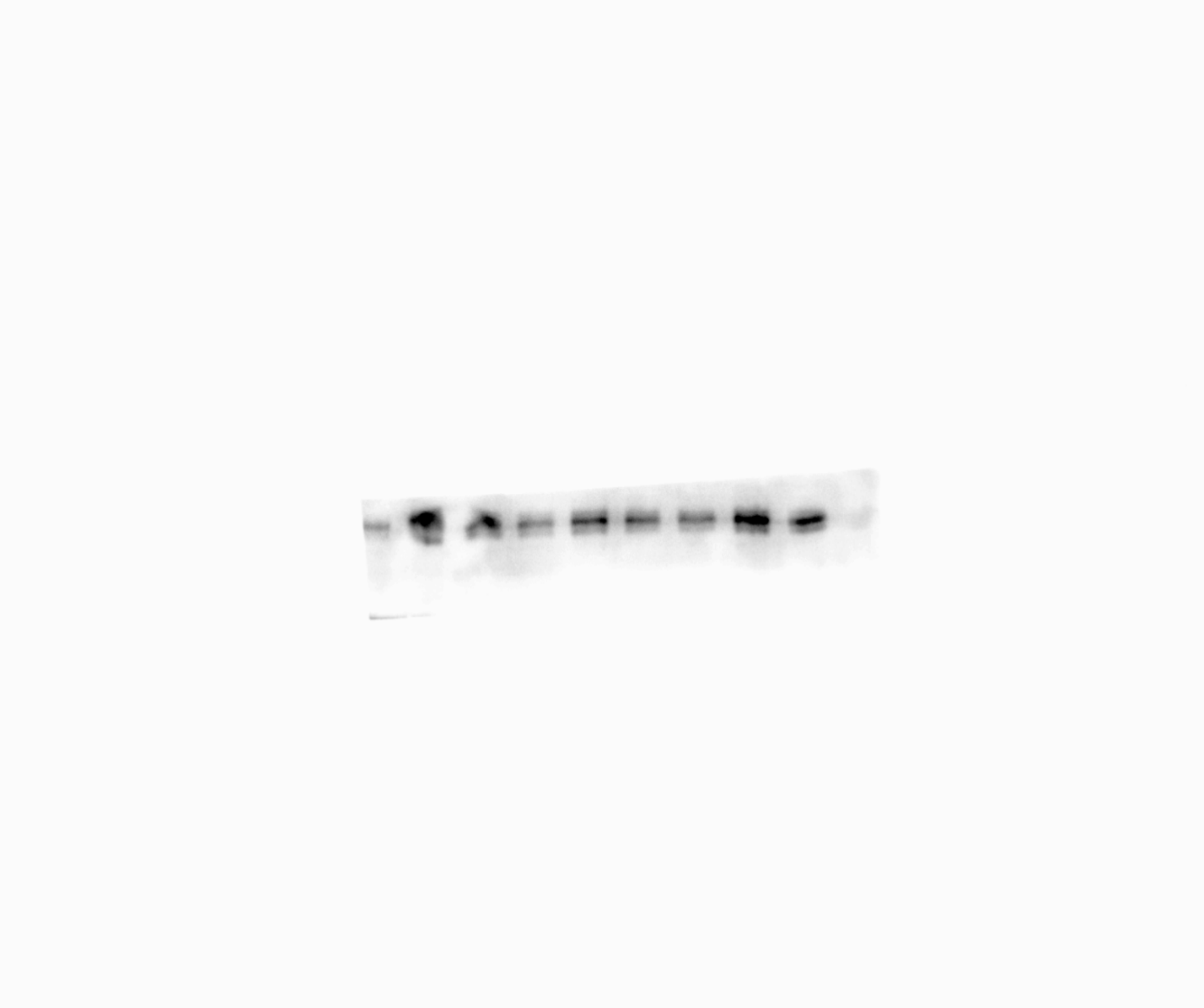


55kDa

1. **β-actin**

**Nor1# Mod1# WB1# | Nor2# Mod2# WB2# | Nor3# Mod3# WB3#**


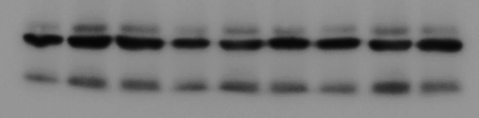


43kDa

43KD

**Supplementary Figure 3: Original western blot images.** Figure 6 showed the results of Nor3#，Mod3#, WB3#.
